# Hemodynamics in bicuspid aortic valve relatives with trileaflet aortic valves compared to normal controls using 4D flow MRI

**DOI:** 10.1186/1532-429X-16-S1-P114

**Published:** 2014-01-16

**Authors:** Susanne Schnell, Alex J Barker, Pegah Entezari, Amir R Honarmand, SC Malaisrie, Patrick M McCarthy, Jeremy Collins, James C Carr, Michael Markl

**Affiliations:** 1 Dept. of Radiology, Northwestern University, Feinberg Medical School, Chicago, Illinois, USA; 2 Northwestern University, Feinberg Medical School, Division of Cardiothoracic Surgery, Chicago, Illinois, USA; 3 Department Biomedical Engineering, Northwestern University, McCormick School of Engineering, Evanston, Illinois, USA

## Background

Bicuspid aortic valve (BAV) is known as the most common congenital anomaly and its complications (ascending aortic aneurysm, dissection) can lead to noticeable morbidity and mortality. There is increasing evidence that, in addition to its genetic background, changes in aortic hemodynamics may play an important role in the development of aortopathy in these patients. Studies have shown that BAV can substantially alter aortic hemodynamics, but it is unclear if the genetic basis can influence aortic properties and 3D blood flow in BAV relatives with normal tricuspid valves. In this study, we evaluated flow dynamics, aorta diameter and geometry in BAV relatives compared to normal volunteers using 4D flow MRI.

## Methods

**Figure 1:**
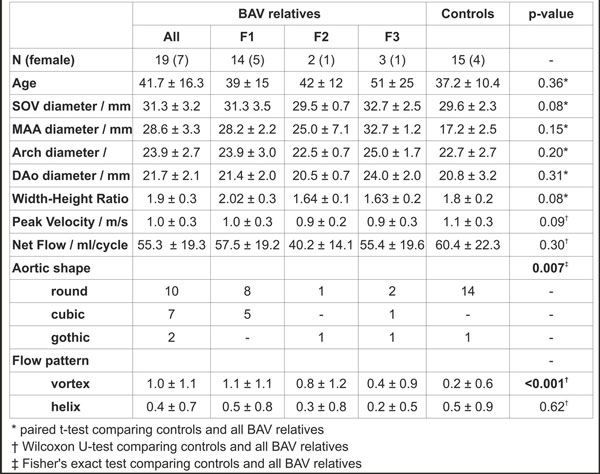
**Demographics, aortic dimensions and hemodynamics of sudy cohort**. All BAV relatives' results are listed in the very left. In addition, the BAV three families (F1, F2, F3) are separately listed. Grading of vortex or helix flow was done as follows: Grade 0: linear flow or flow rotation < 90°, grade 1: rotation of 90 - 180°, grade 2: vertical/helical flow 180 - 360°, grade 3: flow rotation of >360.

15 controls and 3 families with one known BAV case in each family (Figure 1) underwent contrast agent enhanced (BAV relatives: MultiHance, controls: Ablavar) MRI (1.5 or 3T, Siemens, Germany) for the evaluation of ascending aorta (AAo) dimensions, aortic valve morphology, aortic shape, width to height ratio, and aortic 3D blood flow dynamics. To assess valve morphology and global cardiac function, breath-held, ECG-gated time-resolved (CINE) 2D balanced SSFP images were acquired. Aortic blood flow assessment was done using ECG and respiratory-gated CINE 3D phase-contrast MRI with 3D velocity encoding (4D flow) and full volumetric coverage (VENC = 150-250 cm/s, temporal resolution = 38.4-40.0 ms). Net flow and peak velocity were measured at the level of SOV (sinuses of valsalva), AAo (proximal to brachiocephalic trunk), distal aortic arch and descending aorta (DAo, level of left atrium) using commercial software (Ensight, CEI, NC). The presence and severity of helix and vortex flow was assessed by two readers using a defined 3-point grading scale in a blinded manner.

**Figure 2:**
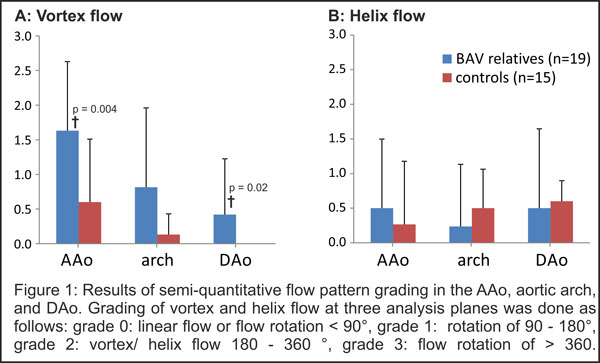
**Results of semi-quantitative flow pattern grading in the AAo, aortic arch, and Dao**. Grading of vortex and helix flow at three analysis planes was done as follows: grade 0: linear flow or flow rotation < 90°, grade 1: rotation of 90 - 180°, grade 2: vortex/helix flow 180 - 360°. Grade 3: flow rotation of > 360.

## Results

Vortex and helix flow grading was performed with excellent inter-observer agreement (Cohen's kappa, κ = 0.73). Vortex flow was significantly more frequent in BAV relatives compared to controls (Figure 2, p < 0.001). Specifically, vortex flow was increased in the AAo (p < 0.004) and DAo (p < 0.025). Helix flow and flow parameters were similar in BAV relatives and controls. 47% of all BAV relatives had a different (p < 0.007) aortic shape than the typical round shape of the controls (93.3%). Peak velocity was reduced (p = 0.009) for gothic aortas and more vortices were found in cubic aortas (p < 0.001).

## Conclusions

The findings of our study demonstrated subtle but significant differences in aortic hemodynamics in BAV relatives compared to age-matched normal controls. Of note, BAV relatives expressed more cubic shaped aortas, which may explain the observed differences. In fact, the significant higher vortex flow in the BAV relatives in the AAo and DAo was related to cubic aortic shape. Future longitudinal studies with larger cohorts matched for aortic shapes are warranted to better understand the dependence of blood flow characteristics on aortic shape, type of valve abnormality and relatives of BAV patients.

## Funding

DFG SCHN 1170/1-1, SIR Foundation pilot study grant, BRACCO.

